# Anti-Invasive and Anti-Proliferative Synergism between Docetaxel and a Polynuclear Pd-Spermine Agent

**DOI:** 10.1371/journal.pone.0167218

**Published:** 2016-11-23

**Authors:** Ana L. M. Batista de Carvalho, Paula S. C. Medeiros, Francisco M. Costa, Vanessa P. Ribeiro, Joana B. Sousa, Carmen Diniz, Maria P. M. Marques

**Affiliations:** 1 Unidade de I&D Química-Física Molecular, Department of Chemistry, University of Coimbra, Coimbra, Portugal; 2 Laboratory of Pharmacology, Department of Drug Science, Faculty of Pharmacy, University of Porto, Porto, Portugal; 3 LAQV/REQUIMTE, Faculty of Pharmacy, University of Porto, Porto, Portugal; 4 Department of Life Sciences, Faculty of Science and Technology, University of Coimbra, Coimbra, Portugal; University of South Alabama Mitchell Cancer Institute, UNITED STATES

## Abstract

The present work is aimed at evaluating the antitumour properties of a Pd(II) dinuclear complex with the biogenic polyamine spermine, by investigating: i) the anti-angiogenic and anti-migration properties of a Pd(II) dinuclear complex with spermine (Pd_2_Spm); ii) the anti-proliferative activity of Pd_2_Spm against a triple negative human breast carcinoma (MDA-MB-231); and finally iii) the putative interaction mediated by combination of Pd_2_Spm with Docetaxel. Anti-invasive (anti-angiogenic and anti-migratory) as well as anti-proliferative capacities were assessed, for different combination schemes and drug exposure times, using the CAM assay and VEGFR2 activity measurement, the Matrigel^TM^ method and the SRB proliferation test. The results thus obtained evidence the ability of Pd_2_Spm to restrict angiogenesis and cell migration: Pd_2_Spm induced a marked inhibition of migration (43.8±12.2%), and a higher inhibition of angiogenesis (81.8±4.4% for total length values, at 4 μM) as compared to DTX at the clinical dosage 4x10^-2^ μM (26.4±14.4%; n = 4 to 11). Combination of Pd_2_Spm/DTX was more effective as anti-invasive and anti-proliferative than DTX or Pd_2_Spm in sole administration, which is compatible with the occurrence of synergism: for the anti-angiogenic effect, IC_50(Pd2Spm/DTX)_ = 0.5/0.5x10^-2^ μM *vs* IC_50(DTX)_ = 1.7x10^-2^ μM and IC_50(Pd2Spm)_ = 1.6 μM. In conclusion, the reported effects of Pd_2_Spm on angiogenesis, migration and proliferation showed that this compound is a promising therapeutic agent against this type of breast cancer. Moreover, combined administration of Pd_2_Spm and DTX was found to trigger a substantial synergetic effect regarding angiogenesis inhibition as well as anti-migratory and anti-proliferative activities reinforcing the putative use of Pd(II) complexes in chemotherapeutic regimens. This is a significant outcome, aiming at the application of these combined strategies towards metastatic breast cancer (or other type of resistant cancers), justifying further studies that include pre-clinical trials.

## Introduction

Angiogenesis plays an important role in several physiological processes but can also be altered in some pathophysiologies, in which either an inhibition or an enhancement have been detected. In cancer, excessive angiogenesis favours tumour spread and development, allowing migration of tumour cells and invasion of tissues far from the primary tumour [1]. Particularly for invasive breast cancers, this process has been found to lead to metastasis even after prolonged periods upon surgical removal of the main tumour, due to neoplastic cell dissemination that leads to minimal residual disease (MRD) [2]. Apart from the well known heterogeneity of this type of tumour, this may explain the poor prognosis associated to it [3–5]. Therefore, development of compounds with the ability to restrict angiogenesis may constitute an important adjuvant strategy in chemotherapeutic regimens, particularly in resistant cancers.

Since Rosenberg’s discovery of cisplatin (*cis*-Pt(NH_3_)_2_Cl_2_, cDDP) [6–8] only three platinum(II)-based compounds are approved as drugs for clinical use (cisplatin, carboplatin and oxaliplatin), but they are often associated to severe side effects and acquired resistance, which strongly limit their application in sole administration. Also, they display a very low efficiency against metastatic cancers. In the last two decades, particular attention has been payed to polynuclear Pt(II) chelates with flexible polyamine ligands, aiming at an improved activity. Furthermore, their Pd(II) analogues have arisen as innovative and promising alternatives to Pt(II)-based drugs [9–13], with less severe adverse effects, particularly when using chelating ligands such as polyamines [13–16]. A careful design of palladium complexes may, thus, allow targetting strategies leading to different profiles of drug activity reducing cross-resistance relative to Pt-agents. In addition to these advances, a combined activity of palladium complexes as anti-invasive and anti-proliferative agents, may exploit their potential as a promising anticancer drug. In fact, although cisplatin/Docetaxel (Taxotere^®^, DTX) combinations have been reported to display synergistic anti-proliferative and anti-angiogenic activities against human triple negative breast cancer (TNBC) [17, 18], reports of combining therapies involving improved new-generation metal-based drugs (namely Pd-based) and yielding synergism towards this type of cancer are still to be found. Therefore, this work is aimed at investigating the ability of a Pd(II) complex, the Pd_2_Spm, to restrict invasion, by inhibiting angiogenesis and/or migration, and simultaneosly act as an anti-proliferative agent against a TNBC cell line (MDA-MB-231). In addition, this work also aims to evaluate the putative interaction of Pd_2_Spm compound when combined with DTX challenging the usefulness of this combination as an alternative therapeutic scheme to fight cancer.

## Materials and Methods

### Chemicals

Crystal violet, DMSO (99.9%), DTX (97%), Dulbecco′s Modified Eagle′s Medium–high glucose (DMEM-HG, 4500 mg/L glucose), formalin (10% neutral-buffered formalin, *ca*. 4% formaldehyde), hydrocortisone, paraffin, penicillin/streptomycin (Pen/Strep), PBS, potassium tetrachloropalladate(II) (K_2_PdCl_4_ >99.9%), spermine (N,N´-bis(3-aminopropyl)-1,4-diaminobutane >97%), Sulforhodamine B (SRB, monosodium salt, 0.5% *(w/v)* solution), Trypan blue (0.04% *(w/v)* solution), trypsin-EDTA (0.05% *(w/v)* solution), vascular endothelial growth factor (VEGF) and inorganic salts and acids and organic solvents (of analytical grade) were from Sigma-Aldrich (Sintra, Portugal). SU5416 –semaxanib was from Selleckchem (Deltaclon, Madrid, Spain). FBS was acquired from Gibco-Life Technologies (Porto, Portugal), and BD Matrigel^TM^ from BD Biosciences (Porto, Portugal). The enzyme-linked immunosorbent assay kit and vascular endothelial growth factor receptor 2 (VEGFR2) protein was obtained from Abcam (Cambridge, UK).

### Synthesis of Pd-based compound

Pd_2_Spm synthesis was obatined according to published procedures [19], optimised by the authors [20]. Briefly, 2 mmol of K_2_PdCl_4_ were dissolved in a minimal amount of water, and an aqueous solution containing 1 mmol of spermine was added dropwise under continuous stirring. The reaction was allowed to occur for 24 hours, after which the resulting yellow powder was filtered off and washed with water. Pd_2_Spm was solubilised in PBS and sterile-filtered before its addition to the cells.

### In vivo CAM assay

The chicken embryo chorioallantoic membrane (CAM) assay is a suitable and cost-effective model for monitoring neovascularization [21–23], and was used as an *in vivo* model for angiogenesis, as described elsewhere [24]. Handling and care of chick embryos were conducted according to the European guidelines (Directive 2010/63/EU) on the protection of animals used for scientific purposes in agreement with the NIH guidelines. This study was carried out in strict accordance with the recommendations in the Guide for the Care and Use of Laboratory Animals of the National Institutes of Health. The protocol was approved by the Committee on the Ethics of Animal Experiments of the Faculty of Pharmacy of University of Porto (Permit Number: 25/10/2015). Briefly, the fertilised chicken Ross strain eggs were obtained from Aviliz, Amor, Portugal and were incubated with agitation at 37.5°C in a humidified atmosphere. After 3 days of incubation, 2.5 mL of albumen were removed, in order to detach the shell from developing CAM, and a window was opened in the eggshell to expose the embryo. At this stage, the fertilised eggs were sealed with paraffin and incubation continued until day 9, when the windows were unsealed. Three PBS-soaked and one VEGF-soaked (10 ng/mL) coverslips were then placed in each egg, in direct contact with the CAM, and the openings were sealed again with paraffin. The coverslips were previously sterilised and pre-treated with hydrocortisone (a cyclooxygenase inhibitor), to avoid inflammatory responses. At day 11, the windows were unsealed and two of the PBS-soaked coverslips were treated with Pd_2_Spm (1 to 4 μM), DTX (1x10^-2^ to 4x10^-2^ μM) and Pd_2_Spm/DTX combinations (1 to 8 μM/1x10^-2^ to 8x10^-2^ μM). The remained untreated coverslips were used as controls (PBS and VEGF). After re-sealed the windows, the eggs were incubated until day 13. At this time-point, the eggs were opened and the coverslips (bound to the CAM) were removed and placed in PBS. Euthanasia was carried out by chicken gestation process terminated following Home Office specific guidelines at the maximum of 14 days of incubation. Contrast-phase images were obtained using a Moticam 5 digital camera coupled to a Motic^®^ AE200 inverted microscope (Spectra Services VWR international) (with a 4x magnification). The digital images were analysed using the Angiogenesis Analyser for Fiji [25].

### Determination of VEGFR2 activity

In vitro VEGFR2 tyrosine kinase activity was analysed by using an enzyme-linked immunosorbent assay kit as previously described [26]. Briefly, the assay in 96-well plates, employs an affinity tag labelled capture antibody and a reporter conjugated detector antibody which immunocaptures the analyte in solution. The complex (captured antibody/analyte/detector antibody) is, in turn, immobilised *via* immunoaffinity of an anti-tag antibody coating the well. Pd_2_Spm and DTX were tested separately (at their IC_50_ concentrations, 1.7 and 1.8x10^-2^ μM, respectively), or in combination (at 6x10^-1^/6x10^-3^ μM for Pd_2_Spm/DTX), by incubation with the antibody mixture. Colour development was determined at 450 nm, in an automated microplate reader (Biotek Winooski, USA).

### In Vitro Assays

#### Breast cancer cell culture

The epithelial human breast cancer cell line MDA-MB-231 (human Caucasian triple-negative, claudin-low, breast carcinoma, lacking the oestrogen, progesterone and human epidermal growth factor receptors (ER, PR and HER2)) was purchased from the European Collection of Cell Cultures (Salisbury, UK). The cells were cultured as monolayers at 37°C in a humidified atmosphere of 5% CO_2_. Cultures were maintained in DMEM-HG medium supplemented with 10% (*v/v*) FBS, 1% (*v/v*) penicillin/streptomycin and sodium bicarbonate. Cells were subcultured at 80% confluence, using 0.05% trypsin-EDTA (1x) in PBS.

#### Cell migration assays

Migration of MDA-MB-231 was measured using the Matrigel^TM^ cell invasion assay [27]. Briefly, the inserts (BD Falcon, Enzifarma, Portugal) were coated with 250 μg.mL^-1^ Matrigel^TM^ and placed in a cell incubator for 2 hours. The top chambers were then seeded with 5×10^4^ cells in DMEM-HG medium without FBS along with 4 μM–Pd_2_Spm and 0.01 μM–DTX, either alone or in combination. The bottom chambers were DMEM-HG supplemented with 10% (*v/v*) FBS. After 72 hours, the cells on the top surface of the membrane (non migrating cells) were gently rubbed with a cotton swab moistened with PBS. The cells spreading to the bottom sides of the membrane (invasive cells) were washed with PBS and fixed with cold 4% formalin for 20 min, and stained with crystal violet. Digital images were acquired with a camera coupled to an inverted microscope (Olympus, Portugal) and invasive cells were quantified by manual counting.

### Cell proliferation tests

#### Sole administration

MDA-MB-231 cell cultures were established in 24-well plates (1 mL/well) at a density of 3x10^4^ cells/cm^2^, and were allowed to attach for about 24 hours. Triplicates were treated for different incubation periods (3 independent experiments) with several concentrations of the tested compounds (Pd_2_Spm from 1 to 16 μM and DTX from 1x10^-2^ to 8x10^-2^ μM). DTX was solubilised in DMSO (concentration never exceeding 0.1% (*v/v*)) and diluted in PBS prior to addition to the cell cultures. According to the population doubling time for MDA-MB-231 cells (26 hours, [12]) the 48 and 72 hours time-points after Pd_2_Spm or DTX addition were chosen. At each of these, the growth media was aspirated, the cells were washed and fixed with ice-cold methanol (1% (*v/v*) in acetic acid) and stored at 20°C. After fixation process, cell proliferation was evaluated through the SRB staining assay to obtain the cellular protein content, considered as directly proportional to the cell density [28]. A 0.01% *(w/v)* DMSO solution was always considered as a control.

#### Combined administration

Cells were seeded in 24-well plates (1 mL/well) at a density of 3x10^4^ cells/cm^2^ and were allowed to attach for about 24 hours. Two different drug administration schemes were used: (i) the cells were simultaneously exposed to 1x10^-2^ μM DTX, and 2 or 4 μM Pd_2_Spm or (ii) the cells were pre-treated with DTX (1x10^-2^ μM, 24 hours), after which, the media was removed and the wells washed, fresh DMEM-HG was added and Pd_2_Spm administered (the end-points being counted from this time forward). Drug co-administration interactions can occur, and these were assessed following the method described by Berenbaum [29].

### Statistical analysis

Results were expressed as a percentage of the control and are presented as mean± for *n* experiments performed (at least three independent experiments). Statistical comparisons between groups, at the same time point, were perfomed with one-way ANOVA followed by Dunnett’s post hoc *t*-test. Signficance was accepted at *p* values < 0.05.

Proliferation data were obtained from experiments in which both controls and cultures exposed to the tested compounds were established and processed in parallel. IC_50_ values (relative inhibitory concentration inducing 50% of cell growth) were calculated from dose-response studies for DTX (0 to 8x10^-2^ μM) and Pd_2_Spm (0 to 16 μM) according to [30].

The synergetic effect of Pd_2_Spm/DTX combination was evaluated according to the method of isoboles [31]. An isobologram was plotted for both drugs in order to access if the addition of Pd_2_Spm to DTX produces a response different than an additive one. A line (isobole) was drawn, connecting the points of 50% maximum response for each drug, in the absence of the other compound, allowing to interpolate the Pd_2_Spm/DTX combinations producing a 50% response.

All data analysis was performed with the GraphPad Prism 6 Software (GraphPad Software, La Jolla, CA, USA).

## Results

### Pd_2_Spm anti-invasive effects

**Fig 1:**
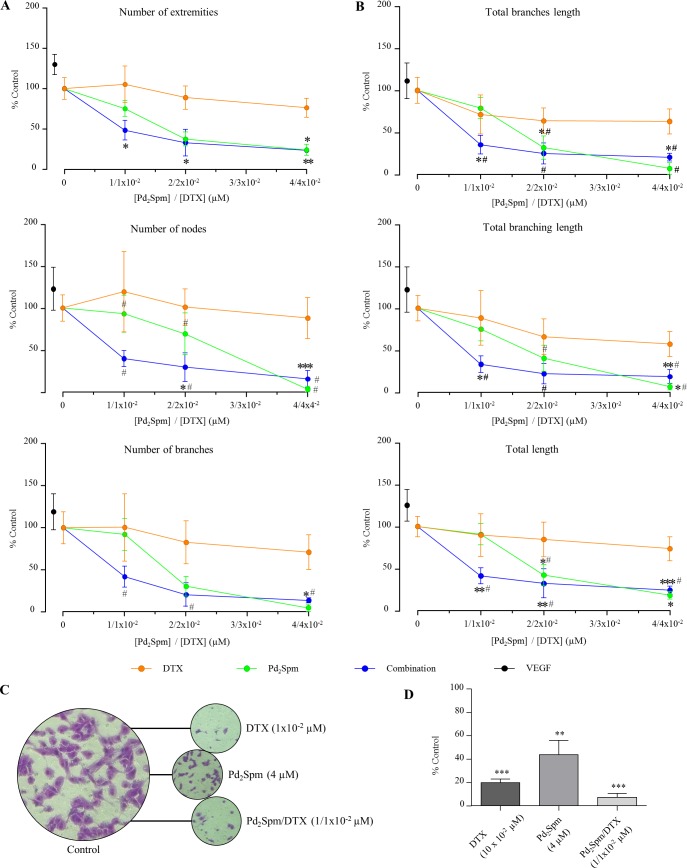
Anti-invasive assays in the presence of DTX, Pd_2_Spm and DTX with Pd_2_Spm combination. Quantitative CAM angiogenesis results in the presence of increasing concentrations of DTX, Pd_2_Spm and Pd_2_Spm/DTX. Eight days after fertilization, DTX, Pd_2_Spm, VEGF (positive control) or PBS (negative control) were added to the coverslip (previously sterilised and treated with hydrocortisone). After incubating the eggs for 48 hours, the CAMs were peeled off and photographed. Digital images were analysed using the Angiogenesis Analyser for Fiji [25]. (A)–number of extremities, nodes and branches; (B)–total branches length, total branching length, total length. Anti-migratory assays for the MDA-MB-231 cell line upon exposure to DTX, Pd_2_Spm and Pd_2_Spm/DTX combination, MDA-MB-231 cell invasion on Matrigel^TM^: (C)–Microscopic image (x10) of MDA-MB-231 cells treated with DTX (1x10^-2^ μM), Pd_2_Spm (4 μM) and Pd_2_Spm/DTX combination (1/1x10^-2^ μM) stained with crystal violet or (D) quantified by simple counting. The results are expressed as a percentage of the control ± SEM. The one-way ANOVA statistical analysis was used, and the Dunnett’s post-test was carried out to verify the significance of the obtained results (*p<0.05, **p<0.01, ***p<0.001 versus the control and #p<0.05 versus the VEGF).

The impact of Pd_2_Spm in angiogenesis modulation was assessed by the CAM assay. Several parameters were counted and evaluated–number of extremities, branches and junctions, as well as total length, total branching length and total branches length. These were found to decrease in a concentration-dependent manner, in comparison to values obtained for both the control (PBS, where normal development of egg neovascularisation occurred) and VEGF conditions (where neovascularisation was stimulated). The effect elicited by increasing concentrations of Pd_2_Spm is depicted in Fig 1.

DTX is a taxane-type drug that may modify angiogenesis [32], and is, thus, currently used for comparison purposes. We have verified DTX-mediated effects on angiogenesis using the CAM assay. Increasing concentrations were tested for DTX in sole administration (1x10^-2^ to 4x10^-2^ μM, corresponding to those commonly used in the clinical practice [33]), and a significant anti-angiogenic effect was observed but only at the highest concentration tested, for all the parameters tested (Fig 1(A) and 1(B)). In turn, Pd_2_Spm-induced inhibition of angiogenesis was very effective: 81.8±4.4% for total length values, at 4 μM, when compared to the 26.4±14.4% DTX-triggered at the clinically used dosage of 4x10^-2^ μM (n = 4 to 11).

The migration ability of the MDA-MB-231 cells was measured using the transwell migration technique (Boyden chamber assay). MDA-MB-231 cells were treated with DTX (1x10^-2^ μM) and Pd_2_Spm (4 μM), for 72 hours. Matrigel^TM^ coated inserts were used to assess whether these treatment schemes affected cancer cell migration and adhesion.

Histological images revealed that Pd_2_Spm (4 μM) and DTX (1x10^-2^ μM) caused a sparse number of cells to spread through the membrane relative to the control, revealing the ability of both compounds per se in preventing cell migration/adhesion (Fig 1).

### Pd_2_Spm anti-proliferative effect

**Fig 2:**
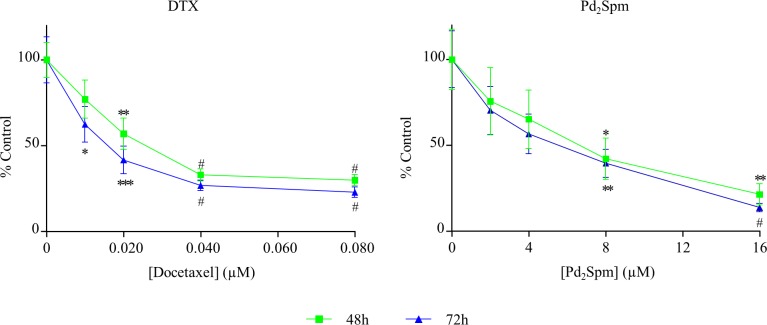
Antiproliferative effect for the MDA-MB-231 cell line upon exposure to Pd_2_Spm and DTX. Simple proliferation of MDA-MB-231 cells:–treated with either DTX (1-8x10^-2^ μM) or Pd_2_Spm (1–16 μM), in sole administration. The results are expressed as a percentage of the control ± SEM. The one-way ANOVA statistical analysis was used, and the Dunnett’s post-test was carried out to verify the significance of the obtained results (*p<0.05, **p<0.01, ***p<0.001, #p<0.0001 versus the control, for the same time-points).

In order to achieve the proposed objectives, confirmation of the high anti-proliferative profile of DTX against the MDA-MB-231 cell line was the next step (Fig 2(A)). Although DTX was not very effective at the lowest concentration used (1.0x10^-2^ μM), for the two highest dosages (4x10^-2^ and 8x10^-2^ μM), it presented a dramatic effect (from 24 hours onwards of exposure). Comparison of the effects of DTX and Pd_2_Spm on the triple-negative breast cancer cells clearly evidenced that while the Pd-agent induced an increasing anti-proliferative activity at all the dosages tested (Fig 2(B)), for DTX a plateau is attained, the highest effect being measured at the 4x10^-2^ μM concentration and unaltered at 8x10^-2^ μM (Fig 2(A)).

Regarding the Pd_2_Spm complex, an IC_50_ value of 1.7 μM at 48 hours was obtained (Table 1), confirming data obtained for the same cell line at a different incubation time, as previously reported [12]: Pd(II) complex exerted a higher cytotoxic effect than that exhibited by cisplatin (at the same concentration range), not only against the oestrogen-unresponsive MDA-MB-231 cells but also towards the oestrogen-responsive MCF-7 cell line.

**Table 1:** IC_50_ values for Pd_2_Spm, DTX and Pd_2_Spm/DTX, and Pd_2_Spm/DTX synergetic effect towards the MDA-MB-231 cell line, evaluated by the SRB method.

	IC_50_	
Experiment	48 hours	72 hours
Simple proliferation assay	Docetaxel (μM)	
	1.5x10^-2^	1.0x10^-2^
		Pd_2_Spm (μM)	
	1.7	1.6
Proliferation assay for combined	Pd_2_Spm + DTX (μM)	
Pd_2_Spm/DTX (scheme i)	3.0x10^-1^	1.2
Proliferation assay for combined	Pd_2_Spm (μM)	
Pd_2_Spm/DTX (scheme ii)	2.5x10^-2^ μM	2.4 x10^-2^ μM
Synergism	Effect	
1x10^-2^ μM DTX / 2 μM Pd_2_Spm	Synergism	
1x10^-2^ μM DTX / 4 μM Pd_2_Spm	Synergism	

Scheme (i)–simultaneous exposure to 1x10^-2^ μM DTX, and 2 or 4 μM Pd_2_Spm–and scheme (ii)–pre-treatment with DTX (1x10^-2^ μM, 24 hours), followed by its removal and administration of Pd_2_Spm (2 or 4 μM). The IC_50_ values were calculated for each drug alone or in combination, according to [30]. Synergism between Pd_2_Spm and DTX was accessed following the isoboles method [31]

### Combination of Pd_2_Spm/DTX as a therapeutic strategy

**Fig 3:**
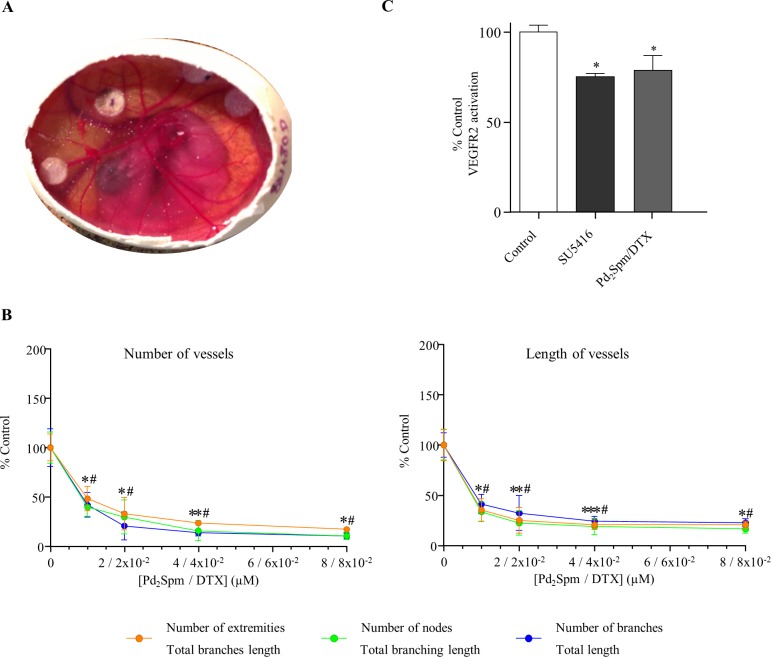
Combination effect of DTX and Pd_2_Spm on angiogenesis. Eight days after fertilization, Pd_2_Spm/DTX, VEGF (positive control) or PBS (negative control) was added to the coverslip (previously sterilised and treated with hydrocortisone). After incubating the eggs for 48 hours, the CAMs were peeled off and photographed. Digital images were analysed using the Angiogenesis Analyser for Fiji [25]. (A)–representative digital CAM image; (B)–quantitative CAM angiogenesis in the presence of increasing concentrations of Pd_2_Spm/DTX; (C) Inhibition of VEGFR2 activity in the presence of IC_50_ concentrations of Pd_2_Spm/DTX (determined by the CAM assay) and SU5416 –semaxanib (VEGFR2 inhibitor). The results are expressed as a percentage of the control ± SEM. The one-way ANOVA statistical analysis was used, and the Dunnett’s post-test was carried out to verify the significance of the obtained results (*p<0.05 versus the control).

Combination therapy schemes intended to enhance cytostatic activity while decreasing the dosage of each individual component, thus leading to reduced acquired resistance and toxicity, are of the utmost importance mainly when synergism is achieved. DTX is an established anti-mitotic taxane-type drug used against several types of cancer (namely TNBC [12], hormone-refractory prostate and lung cancers), but it is generally administered in combination regimes to avoid chemoresistance [34] Pd_2_Spm/DTX combinations were assessed, in search for an additive or synergistic interaction between both drugs (Figs 1 and 3). Their association prompted an increase of the anti-angiogenic effect when compared to the effect observed for each individual compound: 77.7±11.2% for total branching length values versus 42.5±14.9% and 49.5±11.5% for DTX and Pd_2_Spm alone, respectively. At lower concentrations of the combined Pd_2_Spm– 1 μM/DTX– 1x10^-2^ μM, a statistically significant reduction in blood vessels development was observed when compared to the results observed in control conditions: above 50% in the number of extremities (51.6±12.3%), nodes (60.1±9.5%) and branches (58.0±12.6%). Similar results were obtained with total branching length (64.6±11.2%), total branches length (67.0±9.8%) and total length (58.7±9.7%) (n = 4 to 11).

An anti-angiogenic synergetic effect between Pd_2_Spm and DTX was identified (Table 2), following the isobole method [31]. An isobologram for the two drugs (Pd_2_Spm and DTX) was analysed, for each parameter (Table 2), unveiling a clear synergism between both drugs.

**Table 2:** Pd_2_Spm/DTX synergetic effect towards the MDA-MB-231 cell line, evaluated by the CAM assay (at 48 hours of exposure).

	IC_50_			Synergism
Parameter Analysed	Docetaxel	Pd_2_Spm	Pd_2_Spm/Docetaxel	Effect
	(x10^-2^ μM)	(μM)	(μM)	
Number of Extremities	1.9	1.3	0.7 / 0.7x10^-2^	Synergism
Number of Nodes	1.9	2.3	0.6 / 0.6x10^-2^	Synergism
Number of Branches	1.9	1.6	0.7 / 0.7x10^-2^	Synergism
Total Branches Length	0.6	1.5	0.5 / 0.5x10^-2^	Synergism
Total Branching Length	1.5	1.5	0.4 / 0.4x10^-2^	Synergism
Total Length	1.7	1.6	0.5 / 0.5x10^-2^	Synergism

The parameters analaysed were obtained from the analysis carried out with the Angiogenesis Analyser for Fiji [25], from CAM digital images. The IC_50_ values were obtained, for each drug alone or in combination, according to [30]. Synergism between Pd_2_Spm and DTX was accessed following the isoboles method for the lower Pd_2_Spm concentration [31].

Furthermore, the effect of Pd_2_Spm and DTX combination on vascular endothelial growth factor receptor 2 (VEGFR2) activation was assessed. Interestingly, the anti-angiogenic activity of Pd_2_Spm/DTX combination (6x10^-1^/6x10^-3^ μM) was similar to those of SU5416 (IC_50_ = 1 μM [26]), used as a positive control and in a concentration corresponding to IC_50_ inhibited kinase activity. (Fig 3C). SU5416, semaxanib is a tyrosine-kinase inhibitor drug with a potent and selective inhibitory activity at the Flk-1/KDR VEGF receptor tyrosine kinase. It targets the VEGF pathway and both, *in vivo* and *in vitro* studies have verified the antiangiogenic potential [35, 36].

Anti-migratory effect mediated by MDA-MB-231 cell line upon exposure to Pd_2_Spm/DTX combination lead to an intense inhibition of cell migration (7.2%). This effect was more pronounced than that elicited by the individual compounds *per se* (either DTX and Pd_2_Spm, Fig 1(C) and 1(D)).

**Fig 4:**
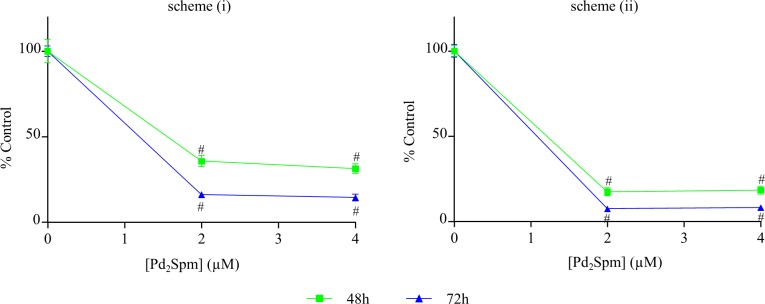
Antiproliferative assays for the MDA-MB-231 cell line upon exposure to Pd_2_Spm/DTX combination. Simple proliferation of MDA-MB-231 cells simultaneously exposed to: (A) 1x10^-2^ μM of DTX, and 2 or 4 μM of Pd_2_Spm (scheme(i)); (B) initially exposed to DTX (1x10^-2^ μM) for 24 hours, and then to Pd_2_Spm (2 or 4 μM) (scheme (ii)). The results are expressed as a percentage of the control ± SEM as a function of Pd_2_Spm concentration (since DTX concentration was the same across all experiments, 1x10^-2^ μM). The one-way ANOVA statistical analysis was used, and the Dunnett’s post-test was carried out to verify the significance of the obtained results (*p<0.05, **p<0.01, ***p<0.001, #p<0.0001 versus the control, for the same time-points).

In turn, and regarding the antitumour activity mediated by combination of Pd_2_Spm and DTX, a high antitumour effectiveness was verified, their growth-inhibiting effect towards the MDA-MB-231 cells having been evaluated through two distinct procedures: (i) the cells were exposed to both drugs at the same time; (ii) the cells were previosly sensitised with DTX– 1x10^-2^ μM and then exposed to two different concentrations of Pd_2_Spm. Fig 4 shows that both schemes led to a higher cytoxicity when compared to each drug alone, which is confirmed by the evaluation of synergism based on cell growth measurements (Table 2). Also, pre-sensitisation with DTX prompted an even more pronounced effect than simple co-administration of both drugs.

## Discussion

In the present work we revealed Pd_2_Spm complex as a promising anticancer drug due to its combined: i) anti-angiogenic, ii) anti-migratory and iii) anti-proliferative activities. Moreover, Pd_2_Spm complex when combined with a well established anticancer drug (taxane) revealead promising synergistic effects, allowing the use of lower concentrations to achieve equivalent anti-invasive and anti-proliferative effects.

The anti-angiogenic activity elicited by different concentrations of Pd_2_Spm complex was compared with that obtained by DTX, also tested in different concentrations. From all the analysed DTX concentrations, in sole administration, 4x10^-2^ μM was found to be the most effective dosage in supressing angiogenesis. The anti-angiogenic capacity elicited by the Pd(II) complex, in turn, was suprisingly high, even for the lowest dosage investigated, 1 μM. The measured anti-angiogenic effect was distinct for the Pd_2_Spm and DTX agents. In our experimental conditions, DTX is unable to supress proliferation of endothelial cells (a required condition to hinder formation of new vessels), which was evidenced by its inability to alter the number of vessel extremities, nodes or branches. Therefore, the DTX anti-angiogenic effect measured can only be assigned to its capacity to inhibit formation of tubular-like structures, reflected in the detected slight reduction of the total branches length, total branching length and total length. By opposition, Pd_2_Spm revealed a high efficacy in supressing both endothelial cell proliferation and tubules development.

In this work, a synergetic effect was observed for Pd_2_Spm/DTX combined administration, leading to an effective anti-angiogenic role regarding endothelial cell invasion and migration, as well as tubules formation–this is, therefore, only achieved in the presence of both compounds simultaneously (the taxane and the metal complex). This high anti-angiogenic ability is well justified in view of the recognised effect of the Pd(II) agent on the cellular protein content and the cytoskeleton, previously reported by the authors [12] and closely related to the prevention of formation of the tubular network needed for new vessel development [37] (i.e., preventing one of possible pathways of angiogenesis).

It is well known that angiogenesis involves multiple signalling events (proliferation, migration and vascular permeability), mediated by several pathways. One of such is the VEGFR2 tyrosine kinase activation. In the present study theVEGFR2 activity was determined in the presence of the compounds under study (both isolated and in combination) allowing to conclude that combination of Pd_2_Spm/DTX was able to produce similar inhibitory effect than that elicted by the selective inhibitor for VEGFR2 receptor, the compound SU5416 [35, 36]. It is also concluded that angiogenesis inhibition ascribed to Pd_2_Spm/DTX combinations (in the CAM assay) can be partially endorsed to VEGFR2 tyrosine kinase activation, since the Pd_2_Spm/DTX IC_50_ dosage caused only a 25% inhibition of the VEGFR2 pathway. Indeed, the antiangiogenic activity observed (due to the action of Pd_2_Spm/DTX) can also be explain by the ability of Pd to react with ROS (which have proangiogenic properties) reducing their accumulation in the cells, in a mechanism similar to that occurring with Ni-complex agents [38]. Another possibility lies on the fact that Pd(II) agent can target the cytoskeleton, mostly the microtubules on the cellular protein content and the cytoskeleton (previously reported by the authors [12]), and closely related to the prevention of formation of the tubular network needed for new vessel development [37]. Nevertheless, we cannot exclude the possibility that Pd_2_Spm compound can alter angiogenesis also by modifying other pathways (involved in angiogenesis) such as activation of NRP1 or the ANG (angiopoietin)1/2/TIE 2 receptors [39, 40].

The reported data confirms previous results that describe DTX´s anti-angiogenic effect in combination with other drugs (*e*.*g*. monoclonal antibodies such as the VEGF blocker Bevacizumab, but not metal-based agents). In fact, anti-angiogenic agents have been essentially used in combined administration with established cytotoxic chemotherapies. This is due to the fact that by normalising the vasculature around the tumour the concentration of the chemotherapeutic agent in the diseased tissue is increased.

Regarding anti-invasive and anti-angiogenic abilities, the results obtained clearly evidenced that DTX displayed a considerable anti-invasive capacity *per se*, Pd_2_Spm was also able to hinder cell migration and invasion and the combined administration of Pd_2_Spm/DTX (at 1x10^-2^ μM) showed to be very efficient as an anti-invasive strategy, and significantly more effective than the effect elicited by the taxane in sole administration. The concomitance of this synergetic effect with that detected for anti-proliferative capacity is of paramount relevance for a potential application of these compounds as effective antitumour agents, since their combination schemes (at the dosages and incubation times presently assessed) appear to couple both growth inhibiting and anti-invasive activities against triple negative breast carcinoma.

Indeed, this polynuclear Pd(II) complex is therefore shown to be a very promising antitumour agent, corroborating previous studies by the authors published by our group in other cells lines such as MCF-7 [12] reporting a high impact on proteins and DNA backbone [41] as compared to its Pt(II) homologue and cisplatin, as well as a significant effect on cell morphology particularly targetting the cytoskeleton (mostly the microtubules, [12]), that may explain, at least in part, the anti-angiogenic effect presently observed associated to the inhibition of tubular-like structure formation [37]: these compounds have been shown to display a higher cytotoxicity towards breast cancer (namely TNBC [12]), due to a more severe DNA damage via long-range interstrand drug–DNA adducts not available to conventional drugs [42].

Combination chemotherapy regimens using metal-based agents (*e*.*g*. cisplatin-like complexes) plus DTX were found to be highly efficient for improving the patients´ overall survival, mainly in low-prognosis cancers [43, 44]. Therefore, in the present work, apart from assessing the anti-proliferative capacity of each tested agent separately–Pd_2_Spm and DTX–the Pd_2_Spm/DTX combination was evaluated, at different dosages, and an obvious synergetic activity having been observed leading to much lower IC_50_ values relatively to those ascribed to the Pd(II) complex alone (up to two orders of magnitude, *e*.*g*. 2.5x10^-2^ *vs* 1.7 μM, at 48 hours). Cell pre-sensitisation with DTX (for 24 hours), as opposed to simultaneous drug administation, triggered a considerably more noticeable effect. However, the molecular basis for this sensitisation still remains to be elucidated. Overall, combination between the inorganic agent and the taxane prompted a striking enhancement in cell growth inhibiting efficiency for this type of invasive breast cancer. Also, anti-angiogenic drugs *per se* were shown to have limited survival benefits. Additionally, this marked synergetic effect between Pd_2_Spm and DTX may be justified through the cell sensitisation triggered by the metal-based agent, known to induce a severe DNA damage *via* direct coordination to the purine bases [12, 45]. DTX, as opposed to the DNA-damaging metal-based agents, interferes with cell division by acting on the cytoskeleton. However, the exact mechanism of synergism between these two agents is still to be clarify at the molecular level, and will be the goal of future studies.

Following the studies on combination schemes of Paclitaxel with the monoclonal antibody Bevacizumab, commonly used as a first-line therapy against metastatic breast cancer [46], the present study evidences the potential of combined administration of another taxane drug (DTX) with a Pd(II)-based agent (Pd_2_Spm) that has previously displayed promising cytotoxic properties against human invasive breast cancer, combined with less severe deleterious side effects [12]. Actually, this strategy couples the high cytotoxicity of the DNA-damaging polynuclear metal complex with the anti-angiogenic capacity of both the taxane derivative and the Pd-drug, turning Pd_2_Spm into a dual cytotoxic and antiinvasive (anti-angiogenic and anti-migratory) compound. Moreover, DTX is suggested to overcome the recognised VEGF-mediated protective role against Pt-drugs´ anti-proliferative activity (firstly reported for cisplatin-treated human ovarian carcinoma [47]), therefore enhancing Pd_2_Spm-induced cell death.

## Conclusions

In conclusion, in the present study, the Pd_2_Spm effects on angiogenesis, migration and proliferation were reported, revealing this compound as a promising therapeutic agent to treat cancer. Moreover, a combined administration of Pd_2_Spm and DTX was assessed and found to trigger a substantial synergetic effect regarding angiogenesis inhibition, but also anti-migratory and anti-proliferative effects, reinforcing the putative use of metal-based Pd(II) complexes in chemotherapeutic regimens. This is a major conclusion, aiming at the application of these combined therapeutic strategies against low prognosis metastatic breast cancer (or other type of resistant cancer), justifying further studies that include pre-clinical trials.

## References

[1] Ferrara N, Kerbel RS. Angiogenesis as a Therapeutic Target. Nature. 2005;438(7070):967–74. 10.1038/nature04483 16355214

[2] Janni W, Vogl FD, Wiedswang G, Synnestvedt M, Fehm T, Juckstock J, et al Persistence of Disseminated Tumor Cells in the Bone Marrow of Breast Cancer Patients Predicts Increased Risk for Relapse—A European Pooled Analysis. Clin Cancer Res. 2011;17(9):2967–76. 10.1158/1078-0432.CCR-10-2515 21415211

[3] Weigelt B, Peterse JL, van 't Veer LJ. Breast Cancer Metastasis: Markers and Models. Nat Rev Cancer. 2005;5(8):591–602. 10.1038/nrc1670 16056258

[4] Ismail-Khan R, Bui MM. A review of triple-negative breast cancer. Cancer Control. 2010;17(3):173–6. 20664514 10.1177/107327481001700305

[5] Krawczyk N, Banys M, Hartkopf A, Hagenbeck C, Melcher C, Fehm T. Circulating tumour cells in breast cancer. Ecancermedicalscience. 2013;7(352):1–29. 10.3332/ecancer.2013.352 24066018 PMC3776645

[6] Rosenberg B, Vancamp L, Krigas T. Inhibition of Cell Division in Escherichia coli by Electrolysis Products from a Platinum Electrode. Nature. 1965;205:698–9. 10.1038/205698a0 14287410

[7] Rosenber B, Vancamp L, Trosko JE, Mansour VH. Platinum Compounds—a New Class of Potent Antitumour Agents. Nature. 1969;222(5191):385 10.1038/222385a0 5782119

[8] Wang D, Lippard SJ. Cellular Processing of Platinum Anticancer Drugs. Nat Rev Drug Discov. 2005;4(4):307–20. 10.1038/nrd1691 15789122

[9] Fiuza SM, Amado AM, Oliveira PJ, Sardao VA, Batista de Carvalho LAE, Marques MPM. Pt(II) vs Pd(II) polyamine complexes as new anticancer drugs: A structure-activity study. Lett Drug Des Discov. 2006;3(3):149–51. 10.2174/157018006776286989

[10] Fiuza SM, Amado AM, Marques MPM, Batista de Carvalho LAE. Use of effective core potential calculations for the conformational and vibrational study of platinum(II) anticancer drugs. cis-diamminedichloroplatinum(II) as a case study. J Phys Chem A. 2008;112(14):3253–9. 10.1021/jp710868p 18331011

[11] Fiuza SM, Amado AM, Dos Santos HF, Marques MPM, Batista de Carvalho LAE. Conformational and vibrational study of cis-diamminedichloropalladium(II). Phys Chem Chem Phys. 2010;12(42):14309–21. 10.1039/c0cp00957a 20871895

[12] Fiuza SM, Holy J, Batista de Carvalho LAE, Marques MPM. Biologic Activity of a Dinuclear Pd(II)-spermine Complex Toward Human Breast Cancer. Chem Biol Drug Des. 2011;77(6):477–88. 10.1111/j.1747-0285.2011.01081.x 21371266

[13] Marques MPM. Platinum and Palladium Polyamine Complexes as Anticancer Agents: The Structural Factor. ISRN Spectrosc. 2013;2013:29 10.1155/2013/287353

[14] Oliveira WXC, da Costa MM, Fontes APS, Pinheiro CB, de Paula FCS, Jaimes EHL, et al Palladium(II) and platinum(II) oxamate complexes as potential anticancer agents: Structural characterization and cytotoxic activity. Polyhedron. 2014;76:16–21. 10.1016/j.poly.2014.03.049

[15] Kapdi AR, Fairlamb IJS. Anti-cancer palladium complexes: a focus on PdX2L2, palladacycles and related complexes. Chem Soc Rev. 2014;43(13):4751–77. 10.1039/C4CS00063C 24723061

[16] Antunovic M, Kriznik B, Ulukaya E, Yilmaz VT, Mihalic KC, Madunic J, et al Cytotoxic activity of novel palladium-based compounds on leukemia cell lines. Anti-Cancer Drugs. 2015;26(2):180–6. 10.1097/cad.0000000000000174 25280061

[17] Fan Y, Xu BH, Yuan P, Ma F, Wang JY, Ding XY, et al Docetaxel-cisplatin might be Superior to Docetaxel-capecitabine in the First-line Treatment of Metastatic Triple-negative Breast Cancer. Ann Oncol. 2013;24(5):1219–25. 10.1093/annonc/mds603 23223332

[18] Tai CJ, Chen CS, Hung CS, Kuo LJ, Wei PL, Chiou JF, et al Bevacizumab Plus Docetaxel and Cisplatin for Metastatic Breast Cancer: A Pilot Phase II Study. Anticancer Res. 2012;32(12):5501–6. 23225458

[19] Codina G, Caubet A, Lopez C, Moreno V, Molins E. Palladium(II) and Platinum(II) Polyamine Complexes: X-Ray Crystal Structures of (*SP*-4-2)-Chloro{*N*-[(3-amino-*κN*)propyl]propane-1,3-diamine-*κN*,*κN*′}palladium(1+) Tetrachloropalladate (2–) (2 : 1) and (*R*,*S*)-Tetrachloro[μ-(spermine)]dipalladium(II) (= {μ-{*N*,*N*′-Bis[(3-amino-*κN*)propyl]butane-1,4-diamine-*κN*:*κN*′}}tetrachlorodipalladium). Helv Chim Acta. 1999;82(7):1025–37. 10.1002/(Sici)1522-2675(19990707)82:7<1025::Aid-Hlca1025>3.0.Co;2-1

[20] Fiuza SM, Amado AM, Parker SF, Marques MPM, Batista de Carvalho LAE. Conformational Insights and Vibrational Study of a Promising Anticancer Agent: the Role of the Ligand in Pd(II)-amine Complexes. New J Chem. 2015;39(8):6274–83. 10.1039/c5nj01088h

[21] DeFouw DO, Rizzo VJ, Steinfeld R, Feinberg RN. Mapping of the microcirculation in the chick chorioallantoic membrane during normal angiogenesis. Microvasc Res. 1989;38(2):136–47. 10.1016/0026-2862(89)90022-8 2477666

[22] Richardson M, Singh G. Observations on the Use of the Avian Chorioallantoic Membrane (CAM) Model in Investigations into Angiogenesis. Curr Drug Targets. 2003;3(2):155–85. doi: 10.3390/ijms13089959 12769641

[23] Lokman NA, Elder ASF, Ricciardelli C, Oehler MK. Chick Chorioallantoic Membrane (CAM) Assay as an *in vivo* Model to Study the Effect of Newly Identified Molecules on Ovarian Cancer Invasion and Metastasis. Int J Mol Sci. 2012;13(8):9959 10.3390/ijms13089959 22949841 PMC3431839

[24] Ribatti D. Chicken Chorioallantoic Membrane Angiogenesis Model In: Peng X, Antonyak M, editors. Cardiovascular Development: Humana Press; 2012 p. 47–57. 10.1007/978-1-61779-523-7_5 22222520

[25] Carpentier G, Martinelli M, Courty J, Cascone I, editors. Angiogenesis Analyzer for ImageJ. 4th ImageJ. User and Developer Conference proceedings; 2012; Mondorf-les-Bains, Luxembourg.

[26] Lu J, Zhang K, Nam S, Anderson RA, Jove R, Wen W. Novel angiogenesis inhibitory activity in cinnamon extract blocks VEGFR2 kinase and downstream signaling. Carcinogenesis. 2010;31(3):481–8. 10.1093/carcin/bgp292 19969552 PMC3105590

[27] Kramer N, Walzl A, Unger C, Rosner M, Krupitza G, Hengstschläger M, et al *In vitro* Cell Migration and Invasion Assays. Mutat Res-Rev Mutat Res. 2013;752(1):10–24. 10.1016/j.mrrev.2012.08.001 22940039

[28] Papazisis KT, Geromichalos GD, Dimitriadis KA, Kortsaris AH. Optimization of the Sulforhodamine B Colorimetric Assay. J Immunol Methods. 1997;208(2):151–8. 10.1016/S0022-1759(97)00137-3 9433470

[29] Berenbaum MC. Application of a New Approach for the Quantitation of Drug Synergism to the Combination of Cis-Diamminedichloroplatinum and 1-Beta-D-Arabinofuranosylcytosine. Cancer Res. 1992;52(16):4558–60. 1643648

[30] Sebaugh JL. Guidelines for Accurate EC50/IC50 Estimation. Pharm Stat. 2011;10(2):128–34. 10.1002/pst.426 22328315

[31] Tallarida RJ. Revisiting the Isobole and Related Quantitative Methods for Assessing Drug Synergism. J Pharmacol Exp Ther. 2012;342(1):2–8. 10.1124/jpet.112.193474 22511201 PMC3383036

[32] Reck M, Mellemgaard A, von Pawel J, Gottfried M, Bondarenko I, Cheng Y, et al Anti-Angiogenic-Apecific Adverse Events in Patients with Non-Small Cell Lung Cancer Treated with Nintedanib and Docetaxel. Lung Cancer. 2015;90(2):267–73. doi: 10.3390/ijms13089959 26415992

[33] Crown J, O'Leary M, Ooi WS. Docetaxel and Paclitaxel in the Treatment of Breast Cancer: a Review of Clinical Experience. Oncologist. 2004;9(Suppl 2):24–32. 10.1634/theoncologist.9-suppl_2-24 15161988

[34] Ghanbari P, Mohseni M, Tabasinezhad M, Yousefi B, Saei AA, Sharifi S, et al Inhibition of survivin restores the sensitivity of breast cancer cells to docetaxel and vinblastine. Appl Biochem Biotech. 2014;174(2):667–81. 10.1007/s12010-014-1125-6 25086926

[35] O'Donnell A, Padhani A, Hayes C, Kakkar AJ, Leach M, Trigo JM, et al A Phase I study of the angiogenesis inhibitor SU5416 (semaxanib) in solid tumours, incorporating dynamic contrast MR pharmacodynamic end points. Br J Cancer. 2005;93(8):876–83. 10.1038/sj.bjc.6602797 16222321 PMC2361651

[36] Hoff PM, Wolff RA, Bogaard K, Waldrum S, Abbruzzese JL. A Phase I study of escalating doses of the tyrosine kinase inhibitor semaxanib (SU5416) in combination with irinotecan in patients with advanced colorectal carcinoma. Jpn J Clin Oncol. 2006;36(2):100–3. 10.1093/jjco/hyi229 16449240

[37] Bayless KJ, Johnson GA. Role of the Cytoskeleton in Formation and Maintenance of Angiogenic Sprouts. J Vasc Res. 2011;48(5):369–85. 10.1159/000324751 21464572 PMC3080587

[38] Zec M, Srdic-Rajic T, Konic-Ristic A, Todorovic T, Andjelkovic K, Filipovic-Ljeskovic I, et al Anti-metastatic and anti-angiogenic properties of potential new anti-cancer drugs based on metal complexes of selenosemicarbazones. Anticancer Agents Med Chem. 2012;12(9):1071–80. 10.2174/187152012803529682 22583413

[39] Carmeliet P, Jain RK. Molecular mechanisms and clinical applications of angiogenesis. Nature. 2011;473(7347):298–307. 10.1038/nature10144 21593862 PMC4049445

[40] Gacche RN, Meshram RJ. Angiogenic Factors as Potential Drug Target: Efficacy and Limitations of Anti-angiogenic Therapy. Biochim Biophys Acta-Rev Cancer. 2014;1846(1):161–79. 10.1016/j.bbcan.2014.05.002 24836679

[41] Batista de Carvalho ALM, Pilling M, Gardner P, Doherty J, Cinque G, Wehbe K, et al Chemotherapeutic Response to Cisplatin-like Drugs in Human Breast Cancer Cells Probed by Vibrational Microspectroscopy. Farad Discuss. 2016;187(0):273–98. 10.1039/C5FD00148J 27063935

[42] Malina J, Farrell NP, Brabec V. DNA Condensing Effects and Sequence Selectivity of DNA Binding of Antitumor Noncovalent Polynuclear Platinum Complexes. Inorg Chem. 2014;53(3):1662–71. 10.1021/ic402796k 24428232

[43] Gralla RJ, Cole JT, Robertson CN, Marques CB, Rittenberg CN. Docetaxel Plus Cisplatin: An Active Combination Regimen in Non-Small-Cell Lung Cancer. Oncology. 1997;11(Suppl 7):27–30.

[44] Roth AD, Maibach R, Martinelli G, Fazio N, Aapro MS, Pagani O, et al Docetaxel (Taxotere®)-cisplatin (TC): An effective drug combination in gastric carcinoma. Ann Oncol. 2000;11(3):301–6. 10811496 10.1023/a:1008342013224

[45] Marques MPM, Gianolio D, Cibin G, Tomkinson J, Parker SF, Valero R, et al A Molecular View of Cisplatin's Mode of Action: Interplay with DNA Bases and Acquired Resistance. Phys Chem Chem Phys. 2015;17(7):5155–71. 10.1039/c4cp05183a 25601325

[46] Nielsen DL, Andersson M, Andersen JL, Kamby C. Antiangiogenic Therapy for Beast Cancer. Breast Cancer Res. 2010;12(5):209 10.1186/bcr2642 21067536 PMC3096961

[47] Hu G, Ryan S, Zhu Y, Reed E, Li X, Wang G, et al Vascular Endothelial Growth Factor Modulates Cisplatin Sensitivity in Human Ovarian Carcinoma Cells. Cancer Therapy. 2003;1:31–7.

